# Development of a Wireless Mesh Sensing System with High-Sensitivity LiNbO_3_ Vibration Sensors for Robotic Arm Monitoring

**DOI:** 10.3390/s19030507

**Published:** 2019-01-26

**Authors:** Yi-Chun Du, David T.W. Lin, Chun-Ping Jen, Choon Wei Ng, Chi-Ying Chang, Ya-Xuan Wen

**Affiliations:** 1 Department of Electrical Engineering, Southern Taiwan University of Science and Technology, Tainan City 71005, Taiwan; ma620223@stust.edu.tw; 2 Institute of Mechatronic System Engineering, National University of Tainan, Tainan City 70005, Taiwan; david@mail.nutn.edu.tw (D.T.W.L.); m10624011@mail.nutn.edu.tw (C.-Y.C.); 3 Department of Mechanical Engineering and Advanced Institute of Manufacturing with High-tech Innovations, National Chung Cheng University, Chiayi County 62102, Taiwan; chunping.jen@gmail.com (C.-P.J.); byhi741@gmail.com (Y.-X.W.)

**Keywords:** multi-axis robots, LiNbO_3_ vibration sensor, wireless mesh network (WMN), sensor node (SN), monitoring system (MS)

## Abstract

In recent years, multi-axis robots are indispensable in automated factories due to the rapid development of Industry 4.0. Many related processes were required to have the increasing demand for accuracy, reproducibility, and abnormal detection. The monitoring function and immediate feedback for correction is more and more important. This present study integrated a highly sensitive lithium niobate (LiNbO_3_) vibration sensor as a sensor node (SN) and architecture of wireless mesh network (WMN) to develop a monitoring system (MS) for the robotic arm. The advantages of the thin-film LiNbO_3_ piezoelectric sensor were low-cost, high-sensitivity and good electrical compatibility. The experimental results obtained from the vibration platform show that the sensitivity achieved 50 mV/g and the reaction time within 1 ms. The results of on-site testing indicated that the SN could be configured on the relevant equipment quickly and detect the abnormal vibration in specific equipment effectively. Each SN could be used more than 10 h at the 80 Hz transmission rate under WMN architecture and the loss rate of transmission was less than 0.01% within 20 m.

## 1. Introduction

Smart factories have inevitably been part of the global trends, and Industry 4.0 proposed by Germany is recognized as a specific example. Industry 4.0 will combine the Internet-of-Things (IoT), cloud platforms, big data, and smart manufacturing to make manufacturing production more intellectualized and automated, creating a new generation of industrial revolution [1,2]. Moreover, many countries have gradually faced the decline in labor force, rising costs, and rising demand for customization with small volume but large varieties. All industries have begun to think about how to intellectualize and automate traditional manufacturing. Within the process of automated production in many industries, multi-axis robots and mobile carriers play an indispensable role, which can be recognized as the key to the success of smart production in the future [3]. In smart production, the accuracy and reproducibility of the product during the production process become higher [4]. In addition, the status determination of the machine or the lifetime prediction of machine usage has become more important. During the period of tight production, the machine will be operating continuously. If there is a case of machine failure in the production line, all the semi-finished products will be affected, so will the entire production line, causing considerable loses. In particular, the production schedule of fully automated factories, and its impact will be even more profound. At present, a factory has achieved the periodic inspections through the Predictive Diagnostic Maintenance System (PDMS), which mostly maintains the machine for a fixed period of time [5]. If the machine failure could be detected at the time and could be informed to the relevant staff of the factory immediately and have the machine repaired as soon as possible. We believe that the loss of the project could achieve a minimum level of conditions.

In order to take control of the status of factory facilities, especially for the non-fixed-cycle real-time monitoring of the robotic arm and mobile vehicle, many researches in recent years have changed the concept of sensor node to use IoT to make implementation of smart monitoring. The IoT architecture can be divided into three levels: the 1st level is effective overall perception, the 2nd level is the effective message transmission, and the 3rd level is the real-time calculation and diagnosis in the cloud. The proposed study referred this kind of architecture to achieve real-time assessment of automated facility as well. In the sensing node of the IoT, it is one of the most commonly used methods to sense the vibration of the robotic arm, the mobile vehicle’s joints, and the drive motor. There are several types of vibration-sensing devices, such as using Fiber Bragg Grating (FBG), Inertial Measurement Unit (IMU) and Linear Velocity Transducer (LVT) to measure the vibration signal and present error message through a relative algorithm [6]. Through the enhancement of device precision, the sensor placement is getting smaller, and the amounts of the placed sensors are increasing. A miniature, highly sensitive, and low-cost sensing element will be an indispensable role.

The vibration piezoelectric sensor applied in the variety field. The quality of the film will affect by the parameters of fabrication, including the concentrations of chemical solutions and annealing temperatures [7]. Crystallization properties of thin films was measured and analyzed in [8]. LiNbO_3_ is one of the most important electrooptical and nonlinear optical single crystals. Its large pyroelectric, piezoelectric, electrooptic, and photoelastic coefficients make it a key material in many different technical applications. LN has a broad homogeneity range with compositions from congruent (cLN, 48.35–48.6 mol% Li_2_O [9]) to stoichiometric (sLN, ~50 mol% Li_2_O [9]). At temperatures above 300 °C the congruent material degrades [10] and is, therefore, inappropriate for high temperature applications. In contrast, our preliminary tests of the stoichiometric crystals indicated that the material is stable up to at least 900 °C [11]. The LiNbO_3_ crystal is a very good piezoelectric transducer material, ferroelectric material, and electro-optic material [12]. The electrical and electromechanical behaviors of LiNbO_3_ as a function of crystal composition, temperature, and oxygen partial pressure have been investigated [13].

Within part of the effective transmission, its existing production lines and facility are normally set up under environment of the traditional factory. However, it is not easy to construct, and to cause job safety anxiety based on adding additional transmission wires or signal adapters. This study used this architecture of wireless mesh network (WMN) to efficiently transmit data from each SN which performs a simple analysis and make an immediate response to the factory.

In terms of wireless network communication, ZigBee has also seen a significant increase in automated industry due to the rise of IoT. ZigBee features a low-power, low-cost, low-data-rate wireless network. Its communication protocol is IEEE 802.15.4 [14,15]. In addition, the data from all the SNs in the factory can be transmitted to the cloud through the wireless and wired network from MS for big data analysis. In accordance with the improvement of the manufacturing process, the data also could be compared with the different production lines that are at the same manufacturing process. The wireless SN designed in this study was a highly adaptable approach in the environment and used a low-power microprocessor with sleep mode function to conserve electricity when the manufacturing process is stopped.

## 2. Materials

**Figure 1:**
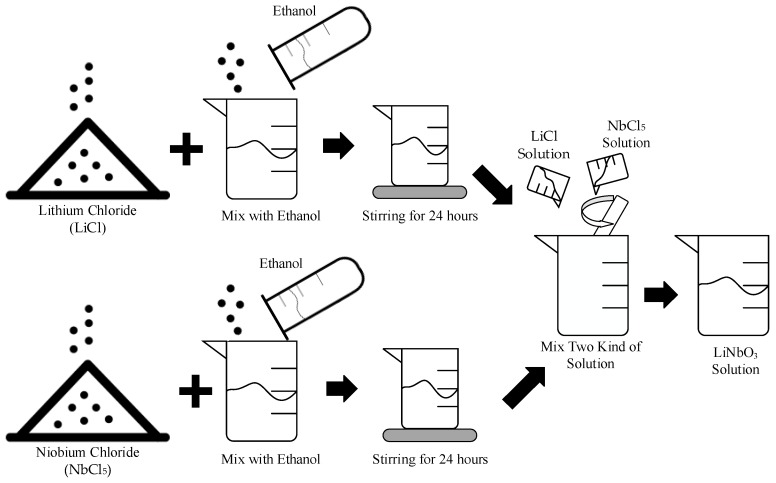
The design of the process of preparation of LiNbO_3_ solution.

**Figure 2:**
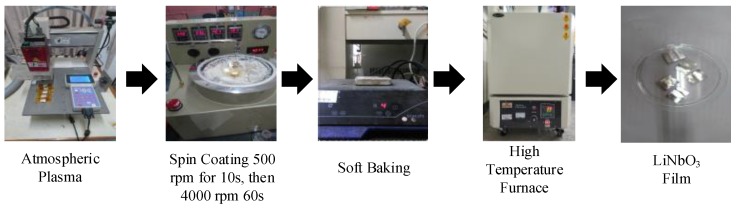
The photographs of fabrication of LiNbO_3_ thin film.

In recent years, the applications of sensors have been growing and maturing. This study was to develop the vibration piezoelectric sensor–LiNbO_3_ thin film deposited on the aluminum substrate. In this work, LiNbO_3_ solution was prepared by the sol-gel method and spin-coating that had the advantages of low cost, easy control of ingredients, and fabricating high purity films at low temperature. The sol-gel solution includes ethanol, lithium chloride (LiCl) and niobium chloride (NbCl_5_). The process of the preparation of LiNbO_3_ solution is shown in Figure 1. Three layers of the film were deposited sequentially. The fabricated processes of the thin film are shown in Figure 2.

### 2.1. Architecture of Sensor Node (SN) Design

**Figure 3:**
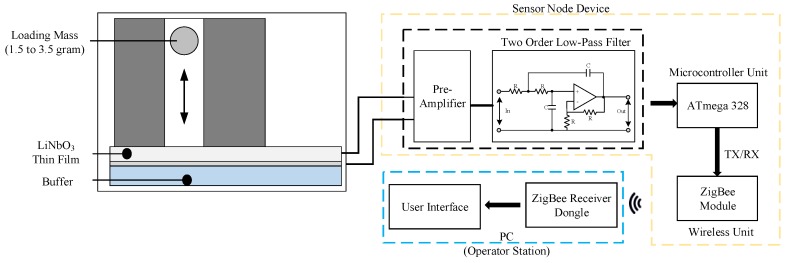
SN architecture.

A novel design for an attachable wireless vibration detector device includes the vibration sensor, the pre-processing and microcontroller unit and the wireless unit. The vibration sensor was made by thin-film LiNbO_3_ and mass printing by a three-dimensional (3D) printer with polylactic acid (PLA) material. The different vibration could produce different signals that go through signal pre-processing to perform voltage stability. Further, the internal micro control unit (MCU) will keep the signal data, and then, the data will be sent by ZigBee wireless module to the operator station for evaluation. Detailed internal functions are shown in Figure 3.

### 2.2. Thin-Film LiNbO3 Sensor

**Figure 4:**
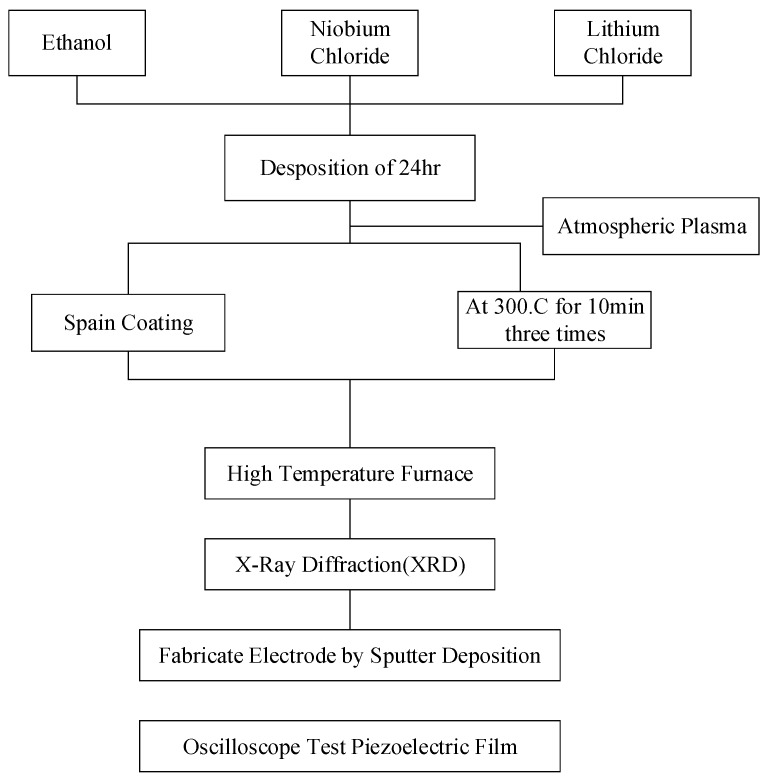
The flowchart of fabrication process.

In this work, lithium niobate solution was prepared by the sol-gel method and spin-coating having the advantages of low cost, easy control of ingredients, and fabricating high purity films at low temperature. The fabrication processes are illustrated in Figure 4.

**Figure 5:**
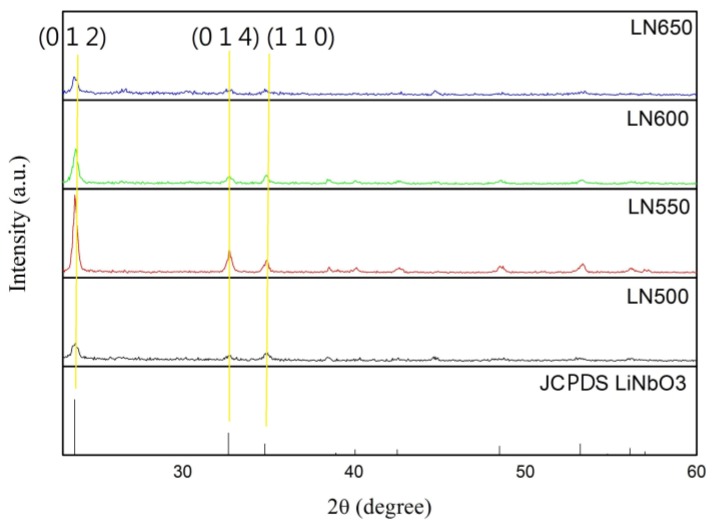
Merge four groups of LiNbO_3_ thin film at different annealing temperature with LiNbO_3_ Joint Committee on Powder Diffraction Standard (JCPDS).

The sol-gel solution includes ethanol, lithium chloride (LiCl) and niobium chloride (NbCl_5_). The molar ratio of LiNbO_3_ was maintained as 0.4 M (Molar concentration). After that, the aluminum substrate was modified by the atmospheric pressure plasma. Then, the prepared acetate solution coated on the flexible aluminum substrate. The parameters of spin coating set as 500 rpm, 10 s and 4000 rpm and 60 s, respectively. Three layers of the film were deposited sequentially. Each layer was baked for 10 min by hot plate under the temperature of 300 °C. The main reason of this procedure was that the organic solvent could be removed to decrease the scorch and crack during the annealed process. Then, the annealing process was preceded under 500 °C, 550 °C, 600 °C and 650 °C. The lattice of film could be rearranged and eliminated the residual stress in this annealing process. Finally, the thin film with better crystalline phase and surface morphology on flexible substrate could be characterized by XRD as shown in Figure 5.

### 2.3. Hardware Design

**Figure 6:**
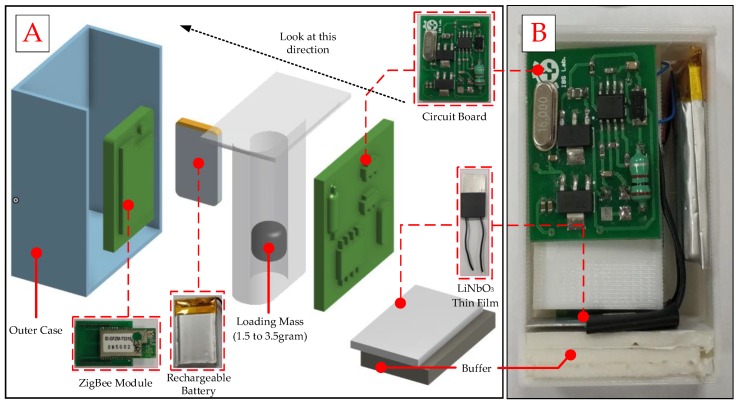
(**A**) The Photograph of SN design and (**B**) the picture of device.

In order to improve ZigBee wireless network limitations in multi-robotic monitoring, this study used an MCU (ATmega328, Microchip Technology, Chandler, AZ, USA) and a ZigBee wireless transmission module (GFZM-T5310, Texas Instruments, Dallas, TX, USA) for vibration detection and wireless transmission. About the MCU module, it includes a power management unit and microcontroller unit. This module has the advantages of low voltage requirement of only 1.8–5.5 V and small size (34 mm × 19 mm) as shown in Figure 6. It also supports real time counter (RTC) for sleep mode and low-power deep sleep mode, which could extend battery life.

The outer case of the device was produced by 3D printing with polylactic acid material, with size measurement was 45 mm × 44 mm × 16 mm as shown in Figure 6. The ZigBee wireless module was used to replace the traditional USB data cable. Further, it could directly attach on robotic arm by magnet. When the abnormal signal was detected, the operators could be informed immediately.

### 2.4. Architecture of WMN Sensing

**Figure 7:**
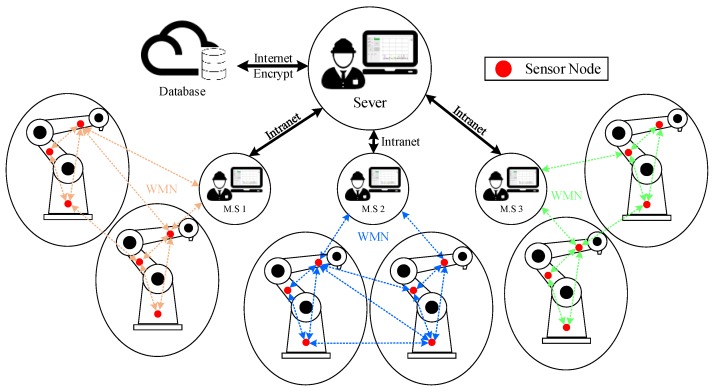
Architecture of WMN.

Our system is designed to apply a real-time MS for automated production lines. Through this system, it was possible to detect immediately whether the mechanical facility on the production line was abnormal. The network architecture was a mesh architecture, which can effectively reduce the difficulty of signal transmission during field deployment [16]. If the abnormal condition happens at any SN in the production line, the warning signal will transmit it to MS through the WMN. It could allow the operator to immediately perform maintenance to reduce the error rate of the production process and problems caused by improper operation. At the same time, through the interface of this system, it can directly show which device has problem, as shown in Figure 7.

Mesh Topology refers to the transmission of data and command control between nodes in the network through dynamic methods. This type of network can maintain the integrity of the connections between nodes and ensure the reliability of data transmission when the number of nodes was large enough. In addition, it was also possible to learn the status of the relatively remote device by means of a jump-through method, which can reduce the one-time wireless transmission power and avoid possible interference with the device. By this way, robotic arm information can be digitized to save manpower costs and reduce work safety accidents.

**Figure 8:**
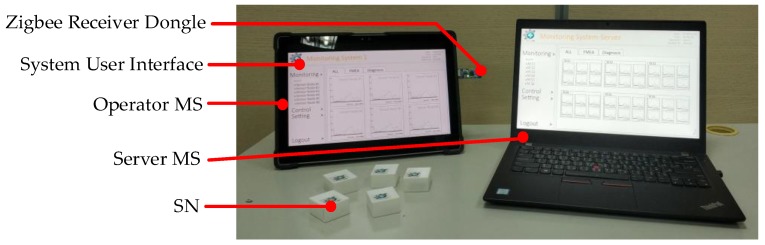
The photograph of the proposed system.

In order to allow operators to monitor multiple machines with SNs at the same time, this study designed a vibration signal that can be monitored immediately. Through the MS interface, instant record and display can be completed by the system. Figure 8 shows the photograph of the proposed system and the user interface. The Zigbee receiver dongle used ZigBee Adapter (CC2530, Texas Instruments, Dallas, TX, USA). The output signal was smooth by five-point moving average.

## 3. Materials and Methods

### 3.1. Sensor Sensitivity Test by Vibration Platform

**Figure 9:**
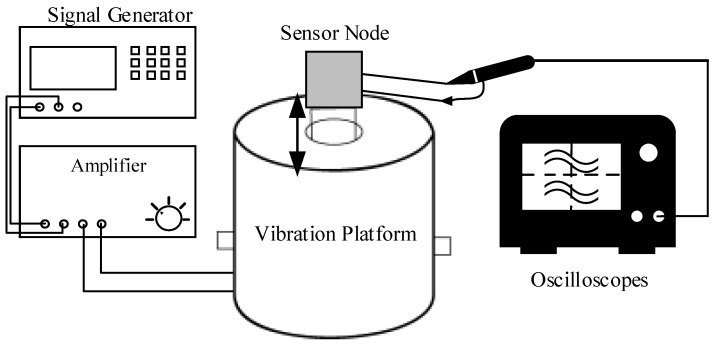
The experimental architecture of sensitivity test.

A facility on the production line might have various unclear vibration factors during operation. Therefore, in this study, the vibration frequency of each mode was simulated with a vibration platform to verify the feasibility of the device on automation facility. The Architecture of the experiment is shown in Figure 9. The vibration platform used in this experiment was Signal Force, which must control the vibration frequency through the signal generator.

In order to simulate the vibration of the on-site machine, in this experiment, SNs with different specifications were prepared for the vibration experiments. The specification including sensor length and loading mass of SNs for the study had three types (A, B and C). The loading mass of type A, B and C was 1.5 g, 2.5 g, and 3.5 g, respectively, and sensor length of each type was 3.5 cm. This experiment through the vibration platform outputs 1 Hz, 3 Hz, 5 Hz vibration. This experimental design referred to previous researches [17,18].

### 3.2. Wireless Signal Test of WMN

**Figure 10:**
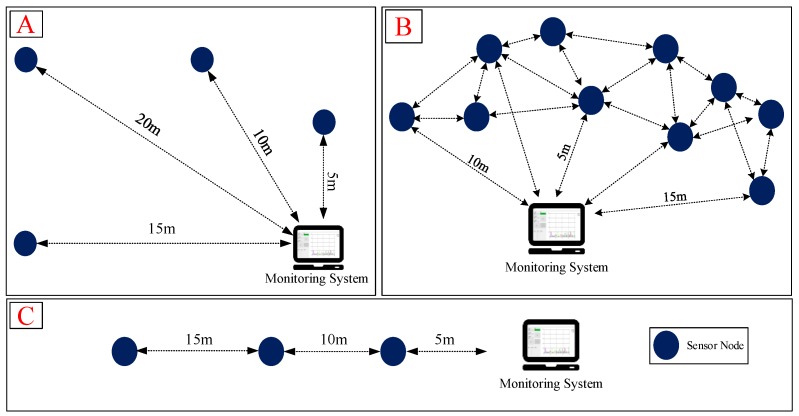
Outdoor wireless test of (**A**) Point-to-Point; (**B**) Mesh and (**C**) End-to-End modes.

In the dreadful production environment, data leakage might occur when data is transmitted, and thus, the expected data integrity could not be achieved. Therefore, our experiment designed a peer-to-peer and mesh network structure to conduct multiple connection tests outdoors. The distance test of wireless transmission is shown in Figure 10. Experiment A was a point-to-point mode with four SNs, B was a mesh mode with ten SNs, and C was an end-to-end mode with four SNs. Transmission distances of experimental B and C between the SNs do not exceed 15 m. The experiments were consistent with the previous research architecture [19,20].

- Experiment A: This experiment mainly tests the relationship between the transmission distance of a single sensing point and the stability of data transmission. The SN transmission frequency was 80 Hz, and the distances were 5 m, 10 m, 15 m, and 20 m. The test time was 1 h and the total amount of data was 1.152 million.
- Experiment B: This experiment mainly tests the transmission distance and stability under the WMN architecture. The testing distance of each SN was no more than 15 m. The transmission rate of the entire system was 800 Hz, and each transmission frequency was 80 Hz in the ten SNs. The test time also recorded 1-h data and the total amount of data received was 2.88 million.
- Experiment C: This experiment mainly tests the relationship between the SN transmission distance and stability under the end-to-end network architecture. The SN transmission frequency was 80 Hz, and the distances in between of the SNs was 5 m, 10 m, and 15 m. The test time was 1-h and the total amount of data was 1.152 million.

### 3.3. Battery Life Test of SN Device

The SN device used a small rechargeable battery with specifications of 3.7 V and 230 mAh, which will be conducive to the future device. In order to determine the usage time of SN device, a battery life experiment would be performed and the test would be divided into two experiments. The experiment was SN device continuous data transmission and in standby mode.

### 3.4. On-Site Testing

**Figure 11:**
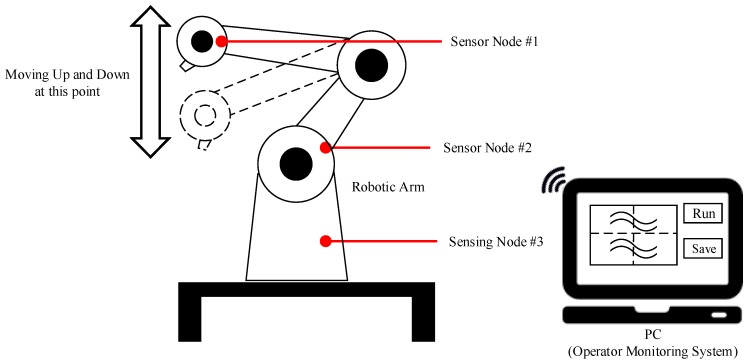
Architecture of On-Site Testing.

After completing the micro-vibration platform simulation experiment, in order to verify the accuracy of the system, this study executed on-site testing with the robotic arm (TVL 500, Toshiba Machine Co., Ltd., Tokyo, Japan) in Streber-Tech Co., Ltd. in Kaohsiung, Taiwan. The architecture diagram is shown in Figure 11. We attached three SNs to the robotic arm to monitor vibration signals, and the red dot shown in the Figure 11 was the position. Then, the operator was required to allow the robot arm to operate normally.

## 4. Results

### 4.1. Sensor Sensitivity Test Results

**Figure 12:**
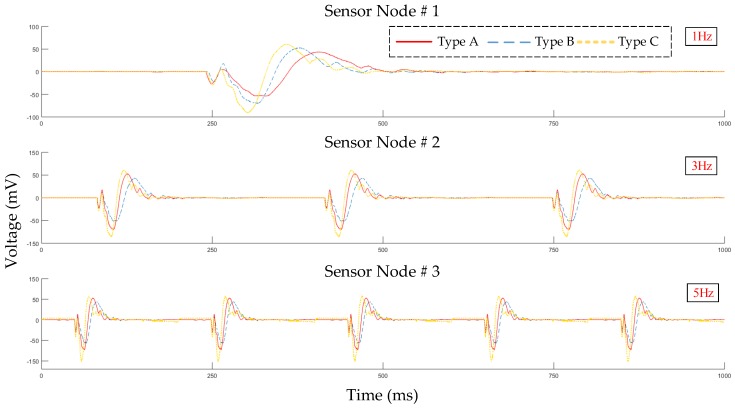
Result signals of oscilloscope from each SN type.

The sensitivity of each SN found that the minimum vibration sensitivity was 50 mV/g, shown in Table 1. The equation of mV/g was representative of the number of millivolts. This does assuage the structural integrity of a specific area as decided by the voltage input into it. In the case of these SN units, the amount was 100 mV. Figure 12 shows the result signals of each SN type that measured by the oscilloscope on the vibration platform.

### 4.2. Wireless Signal Test Results

Experiment A: According to the experimental test results, when the distance exceeded 20 m, the Received Signal Strength Indicator (RSSI) was lower than −88 dBm. The quantity of data loss was 120 in the total 1.152 million data, so the loss rate was about 0.01%. The results are shown in Table 2.

Experiment B and C: The results found out that the transmission loss rate was zero in this experimental design. The result B indicated the wireless transmission of the proposed system was reliable if the maximum distance between two SNs was within 15 m. In most factories, 15 m is enough for system setup, but it is still affected by EMI/EMC interference on-site.

### 4.3. Battery Life Test Results

Experimental results showed that the ATmega328 has higher power consumption in continuous Zigbee transmission, but without continuous transmission, the power consumption is acceptable [21,22]. The results indicated that the SN could be operated up to 10 h with 80 Hz transmission rate. If under the standby mode the battery life could extend to more than 4 days depending on the transmission distance between the SN and MS. 

### 4.4. On-Site Testing Results

**Figure 13:**
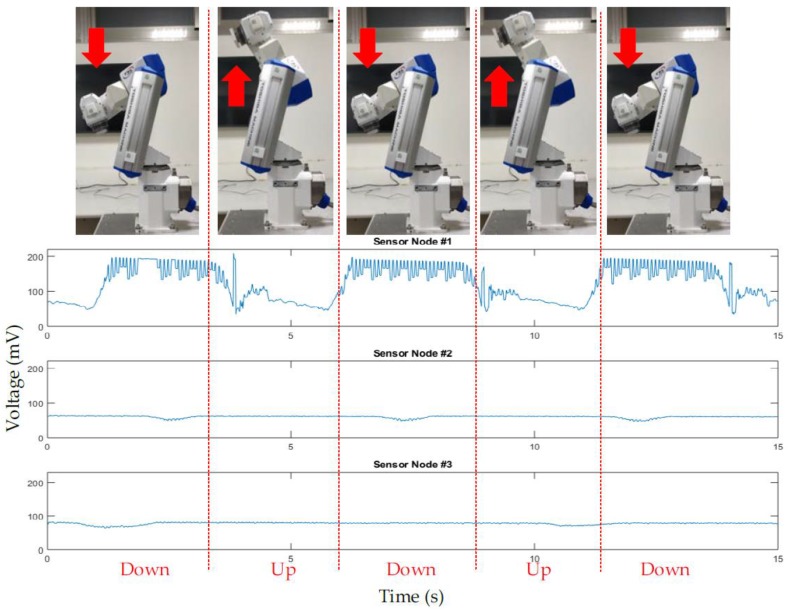
The results of on-site system testing.

According to the results of the on-site test, as the robotic arm moved up and down, we could clearly see that the sensor node #1 signal was obvious because it was the point where the robotic arm moved the most. The sensor node #2 and #3 were static, but these two SNs would still be affected by micro-vibration when the robotic arm was moving, as shown in Figure 13. 

## 5. Conclusions

This research successfully developed a high-sensitivity LiNbO_3_ vibration sensor and integrated it into a low-power SN device with RTC function. With the integration with SNs and WMN architecture, we successfully implemented the IoT monitoring system for robotic arms in automated facilities. According to the results, the SN has not only the same performance with other commercial sensors but also the advantages in low-cost, high-sensitivity, and good electrical compatibility. Therefore, by using the SN of the present study, the maintenance can be performed as soon as possible to assist the factory to analyze abnormal vibration signals of robotic arms. Multi-points and real-time vibration analyses can also be used to predict the life cycle of the robot arm and reduce the possible risk of the production line. In the future, the present study can combine failure mode and effect analysis (FMEA) function to establish an analysis tool to indicate the severity of the failure and the level of occurrence. The proposed system, combined with the FMEA function, is highly potential to be a PDMS that could improve the factory productivity, reduce maintenance costs, and increase the lifetime of robotic arms. In addition, the power management circuit of the piezoelectric energy harvester function could also be consideration. It can produce more power with the same input vibration to extend the use time of the SN [23,24].

## Figures and Tables

**Table 1:** Vibration testing output.

	Type	A	B	C
Frequency (Hz)				
1		50	79	101
3		88	117	129
5		102	124	134

Output Unit: mV.

**Table 2:** Data Transmission in WMN of experiment A.

Distance (m)	RSSI (dBm)	Data Loss (%)
5	−66	0%
10	−71	0%
15	−82	0%
20	−88	0.01%
